# Fully Human Monoclonal Antibody Inhibitors of the Neonatal Fc Receptor Reduce Circulating IgG in Non-Human Primates

**DOI:** 10.3389/fimmu.2015.00176

**Published:** 2015-04-23

**Authors:** Andrew E. Nixon, Jie Chen, Daniel J. Sexton, Arumugam Muruganandam, Alan J. Bitonti, Jennifer Dumont, Malini Viswanathan, Diana Martik, Dina Wassaf, Adam Mezo, Clive R. Wood, Joseph C. Biedenkapp, Chris TenHoor

**Affiliations:** ^1^Dyax Corp., Burlington, MA, USA; ^2^Syntonix Pharmaceuticals (a wholly-owned subsidiary of Biogen Idec.), Waltham, MA, USA; ^3^Biogen Inc., Cambridge, MA, USA

**Keywords:** neonatal Fc receptor, FcRn, phage display, IgG, antibody engineering, autoimmune disease

## Abstract

The therapeutic management of antibody-mediated autoimmune disease typically involves immunosuppressant and immunomodulatory strategies. However, perturbing the fundamental role of the neonatal Fc receptor (FcRn) in salvaging IgG from lysosomal degradation provides a novel approach – depleting the body of pathogenic immunoglobulin by preventing IgG binding to FcRn and thereby increasing the rate of IgG catabolism. Herein, we describe the discovery and preclinical evaluation of fully human monoclonal IgG antibody inhibitors of FcRn. Using phage display, we identified several potent inhibitors of human-FcRn in which binding to FcRn is pH-independent, with over 1000-fold higher affinity for human-FcRn than human IgG-Fc at pH 7.4. FcRn antagonism *in vivo* using a human-FcRn knock-in transgenic mouse model caused enhanced catabolism of exogenously administered human IgG. In non-human primates, we observed reductions in endogenous circulating IgG of >60% with no changes in albumin, IgM, or IgA. FcRn antagonism did not disrupt the ability of non-human primates to mount IgM/IgG primary and secondary immune responses. Interestingly, the therapeutic anti-FcRn antibodies had a short serum half-life but caused a prolonged reduction in IgG levels. This may be explained by the high affinity of the antibodies to FcRn at both acidic and neutral pH. These results provide important preclinical proof of concept data in support of FcRn antagonism as a novel approach to the treatment of antibody-mediated autoimmune diseases.

## Introduction

Autoimmune diseases arise from abnormal immune responses against substances and tissues normally present in the body. More than 150 disorders have been classified as autoimmune or autoimmune-related, affecting 5–8% of the general population (1). In some disorders, IgG autoantibodies are the pathogenic agents of disease and exert their effect through a variety of mechanisms. These include neutralizing the activity of endogenous molecules, fixing of complement and activation of signaling cascades to trigger cell lysis and phagocytosis, and formation of immune complexes which instigate inflammation and interfere with organ function. In addition, antibody responses to biologic replacement therapies such as those used in the treatment of hemophilia and lysosomal storage diseases result in clinical complications and interfere with treatment (2–4).

Traditionally, there have been two main treatment strategies for antibody-mediated autoimmune diseases: immunomodulatory and immunosuppressive therapies. Corticosteroids and small molecule immunosuppressant agents have historically been first-line therapies but in recent years, biologics including B-cell depletion drugs such as rituximab and ofatumumab (5), plasma cell depletion drugs such as bortezomib (6), and complement inhibitors such as eculizumab (7, 8), have been used to treat a variety of autoimmune diseases. While providing clinical benefit, many of these drugs are associated with severe side-effects such as infection that may be life-threatening (9–12). Immunomodulatory therapies include plasmapheresis, intravenous IgG (IVIG), and immunoabsorption (13–19). These therapies are typically used to control disease activity in the acute phase of the disease through dilution or removal or reduction of serum autoantibodies. However, these treatment approaches are expensive, require complicated procedures, and carry risk of severe complications (20). Thus, there remains an unmet need for therapeutic strategies with an improved risk/benefit ratio.

Antagonism of the neonatal Fc receptor (FcRn) may represent an alternative approach to the treatment of diseases caused by high levels of pathogenic IgG antibodies (1, 21, 22). FcRn, initially identified as the neonatal receptor responsible for transcytosis of IgG from mother to offspring to confer short-term passive immunity, plays a critical role in IgG and albumin homeostasis throughout adult life (23–26). In addition to its expression in neonatal intestinal epithelial cells, it is expressed in a variety of cell types, including the vascular endothelium and hematopoietic cells (27–30). It is a heterodimeric receptor possessing an MHC class I-like heavy chain and a non-covalently associated light chain β2-microglobulin (β2M) (31). Crystallography and site-directed mutagenesis reveal that FcRn can bind both IgG and albumin on opposite faces of the heavy chain (32–34). Significantly, the FcRn–IgG/albumin interaction is highly pH-dependent, with higher affinity at acidic pH than at neutral pH. This pH-dependent binding is central to the role of FcRn in regulating systemic IgG concentration.

IgG elimination primarily occurs through intracellular catabolism, following fluid-phase (pinocytosis) or receptor-mediated endocytosis (35). When internalized, IgG can bind to FcRn in the acidic environment of the endosome, thereby protecting it from lysosomal degradation. The FcRn–IgG complex is then recycled back to the cell surface where IgG dissociates at physiological pH. In contrast, IgGs and other serum proteins which fail to bind FcRn enter the lysosomal pathway and are degraded. Thus, the pH-dependent binding of FcRn to IgG (and albumin) is the central mechanism underlying the extended serum half-life of these two plasma proteins. The potential to modify the serum half-life of engineered proteins through their interaction with FcRn was quickly recognized and has since been capitalized upon to maximize the pharmacokinetics and bioavailability of therapeutic antibodies and related biologics (36–38).

Previous studies have demonstrated that mice lacking FcRn have accelerated IgG catabolism and are less susceptible to induction of various autoimmune diseases (39–41). It has also been suggested that the therapeutic benefit of IVIG in the treatment of various autoimmune diseases, may result in part from FcRn saturation and enhanced catabolism of endogenous IgG (40, 42) though not in a mouse model of immune thrombocytopenia (43). IgG and peptide antagonists of FcRn have also been developed and administration of these molecules to mice (44–47), rats (48), and non-human primates (45) accelerated IgG catabolism and reduced disease severity in animal models of myasthenia gravis, arthritis, and glomerular disease (46, 48, 49). Taken together, blockade of IgG binding to FcRn increases the rate of IgG catabolism and has the potential to deplete the body of pathogenic immunoglobulin, representing a potentially novel treatment to reduce pathological IgG autoantibodies.

Herein, we describe the discovery and characterization of fully human monoclonal antibodies that bind with high affinity to human-FcRn at both acidic (pH 6.4) and neutral (pH 7.0) (i.e., pH-independent binding). Following administration of anti-FcRn antibody to rodents and non-human primates, significant reductions in circulating IgG with no changes in albumin, IgM, or IgA were observed. In non-human primates, we observed no evidence of immunosuppression, as monkeys maintain the ability to mount IgM/IgG primary and secondary immune responses in the presence of anti-FcRn antibody. Interestingly, the anti-FcRn antibodies had a short serum half-life but caused a durable and extended reduction in IgG levels, such that serum levels of anti-FcRn antibody did not correlate directly with pharmacodynamic activity. Preliminary studies in rodents and non-human primates found the antibody to be well-tolerated. These results suggest anti-FcRn antibodies may represent a novel therapeutic approach to the treatment of IgG antibody-mediated autoimmune diseases.

## Materials and Methods

### Phage display selection

#### Soluble human-FcRn expression and purification

Soluble human-FcRn (shFcRn) was prepared as previously described (45).

#### Antibody expression and purification

Fabs were expressed in *E. coli* and purified using protein A sepharose as described previously (50). Recombinant Fab fragments were reformatted into full-length human IgG1 antibodies (F-allotype) and either produced by transient transfection in HEK 293T cells as described (51), or stably transfected into CHO (Chinese-hamster ovary) cells using the glutamine synthetase expression system (Lonza Biologics), expressed using a fed-batch fermentation strategy, and purified as previously described (52).

#### Phage display selection

Human antibodies against FcRn were identified from an antibody phage display library (53) using biotinylated shFcRn immobilized on streptavidin-coated magnetic beads (Dynal, M280) and cells expressing hFcRn.

(1) For selections against biotinylated shFcRn, biotinylated shFcRn was initially immobilized on streptavidin beads. Prior to the first selection round, the antibody library was depleted on uncoated streptavidin beads by allowing the library phage to incubate at room temperature for 10 min before removing the supernatant containing the phage and proceeding into the first round of selection.

Phage were allowed to bind to immobilized shFcRn in an acidic binding buffer (pH 6), and were then eluted with polyclonal human IgG (Calbiochem, Catalog # 401114) and monoclonal mouse anti-human-FcRn mAb (3B3) in an acidic buffer. After this competitive elution, all remaining bead-bound phage were used to directly infect *E. coli* and the amplified phage output was used as input for next round of selection. Three rounds of selection against immobilized FcRn protein were performed.

(2) Three rounds of selection against hFcRn- transfected cells were carried out with depletion on an untransfected parental cell line. Phage were allowed to bind to cells in an acidic binding buffer (pH 6), and then were eluted with non-specific human IgG and anti-FcRn mAb (3B3) in the same acidic buffer. After this competitive elution, all remaining bead-bound phage were used to directly infect *E. coli* and the amplified phage output was used as input for next round of selection.

#### Primary screening

Phage isolates were screened by ELISA (streptavidin-immobilized shFcRn with detection by anti-M13 coat protein VIII), positive hits were DNA-sequenced, and unique Fabs batch-processed for *E. coli* expression as isolated Fab fragments from the pMID21 vector as described (50).

#### Affinity maturation

An affinity maturation library was constructed from the lead Fab by employing mixed nucleotide synthesis of heavy chain variable complementary determining region 3 (HV-CDR3) where the nominal base was present at 85% and each of the others at 5%. The library repertoire of HV-CDR1 and CDR2 were added to the parent heavy chain for a set of affinity matured variants. The high-affinity variants from HV-CDR1–2 and HV-CDR3 affinity maturation were combined to create a small population for screening of improved variants. A germlined light chain was used to build the affinity maturation library in order to avoid further sequence optimization.

#### Affinity maturation library selection

Library selections were carried out using a combination of cells and protein targets with lower protein targets concentrations in each round for selection of high-affinity variants.

(1) Selections against biotinylated shFcRn: two rounds of selection against biotinylated shFcRn were carried out with depletion on uncoated streptavidin beads as described previously. Phage were allowed to bind to target in acidic binding buffer (pH 6), and were then eluted with parental M90-F11 IgG in a pH 7.4 buffer (parental M90-F11 IgG was the lead anti-FcRn antibody identified during the primary screening campaign). After competitive elution/wash, all remaining bead-bound phage were used to directly infect *E. coli*. The eluted phage output was used as input for the next round of selection. Round 2 output was used in alternate round 3 selection against hFcRn-transfected cells followed by a fourth round selection using biotinylated shFcRn selections using the same elution strategy.
(2) Selections against hFcRn expressing cells: two rounds of selection against hFcRn-transfected cells were carried out. Phage were allowed to bind to cells in acidic binding buffer (pH 6) at 4°C, and were then eluted with parental M90-F11 IgG in a pH 7.4 buffer. After competitive elution/wash, all remaining bead-bound phage were eluted by cell lysis with magnetic streptavidin beads and subsequent infection of bacteria. The eluted phage output was used as input for next round of selection. Two additional rounds of selection against biotinylated shFcRn were carried out as described in (1).

### Surface plasmon resonance analysis of anti-FcRn variants

Surface plasmon resonance (SPR) measurements were performed using a BIAcore 3000 (GE Healthcare). Anti-FcRn variants were immobilized by amine coupling on CM5 sensor chips. To measure the kinetic parameters of anti-FcRn antibody interactions with FcRn analyte, twofold serial dilutions prepared from 100 nM of FcRn were injected in duplicate for 5 min at 50 μl/min with a 15-min dissociation phase. The sensor chip surface was regenerated with a 30-s pulse of 10 mM glycine pH 1.5 at a flow rate of 75 μl/min followed by a 15-s pulse of buffer. Measurements were performed at 25°C using HBS-P (10 mM HEPES, 150 mM NaCl, and 0.005% surfactant P20 at pH 6.0 and pH 7.5) as the running buffer. The reference flow cell was activated and blocked in a mock amine coupling reaction. The data were fit to a 1:1 binding model using BIAevaluation v.4.1 software.

### Experiment 1: IgG catabolism and pharmacokinetics of the anti-FcRn antibody DX-2504 in hFcRn transgenic mice (TG32B)

#### TG32B mice

Transgenic mice expressing hFcRn [“TG32B,” mFcRn (−/−), mβ2M (−/−), hFcRn (+/+), hβ2M (+/+)] were originally obtained from Dr. Derry Roopenian (The Jackson Laboratory). All animal studies were approved by the Institutional Animal Care and Use Committee (IACUC) and performed in accordance with the Guide to the Care and Use of Laboratory Animals.

#### IgG catabolism in transgenic mice

TG32B mice (*n* = 4) received 500 mg/kg hIgG intravenously. Twenty-four hours later, approximately 60 μl of blood was collected by a tail snip prior to dosing the mice with intravenous (IV) or SC DX-2504 (12.21 mg/ml). Additional blood samples were collected from each mouse at 0.5, 24, 48, 72, 96, and 144 h after administration of DX-2504. Serum was prepared and stored at −20° C.

#### hIgG ELISA

Serum levels of human IgG were measured using a specific hIgG ELISA. Briefly, 96 well plates were coated with 50 μl/well goat anti-human IgG (Fab-specific) antibody (Pierce Biotechnology, Rockford, IL, USA, Catalog # 31122) at 4°C overnight. Plates were then blocked with 200 μl/well of 2% bovine serum albumin (BSA) in phosphate buffered saline (PBS) for 1 h at 37°C. Samples and standards (100 μl/well) were diluted in 2% BSA/PBS and incubated at 37°C for 1 h. The standard curve of hIgG ranged from 200 to 1.56 ng/ml in twofold dilutions. Plates were then washed three times with 300 μl/well PBS supplemented with 0.05% Tween-20 (PBST) in a Tecan plate washer before adding 100 μl/well goat anti-human IgG (Fc-specific)-horseradish peroxidase (HRP) conjugate (Pierce Biotechnology, Catalog # 31413) diluted 1:25,000 in 2% BSA/PBS. Plates were incubated with detection antibody for 1 h at 37°C before again washing three times with PBST. Reactions were developed with 100 μl/well TMB substrate (Supersensitive 1-component HRP substrate, BioFX, Owing Mills, MD, USA, Catalog # TMBS-0100-01) at room temperature for approximately 4 min. Reactions were stopped by addition of 100 μl/well 0.25 M sulfuric acid, and plates were read at 450 nm using a Spectramax spectrophotometer (Molecular Devices, Sunnyvale, CA, USA). The standard curve was then used to calculate serum concentration of IgG.

#### Measurement of anti-FcRn antibody DX-2504 in mouse serum

Serum concentration of DX-2504 was measured using a specific hIgG ELISA. Briefly, 96 well plates were coated with 50 μl/well goat anti-human IgG (Fab-specific) antibody (Pierce Biotechnology, Rockford, IL, USA, Catalog # 31122) at 4°C overnight. Plates were then blocked with 200 μl/well of 2% BSA in PBS for 1 h at 37°C. Samples and standards (100 μl/well) were diluted in 2% BSA/PBS and incubated at 37°C for 1 h. The standard curve ranged from 200 to 1.56 ng/ml of DX-2504 in twofold serial dilutions. In addition, a known amount of DX-2504 was spiked into 100% transgenic mouse serum, which was then diluted to correspond to the dilution factor used for unknown samples (e.g., 1:5,000, 1:2,000, etc.). The concentration of spiked DX-2504 in the 100% mouse serum sample varied depending on the final dilution factor of the unknowns, with the objective to choose a spiked concentration that was expected to fall on the linear portion of the standard curve. Plates were then washed three times with 300 μl/well of PBS supplemented with 0.05% Tween-20 (PBST) in a Tecan plate washer before adding 100 μl/well goat anti-human IgG (Fc-specific)-HRP conjugate (Pierce Biotechnology, Rockford, IL, USA, Catalog # 31413) diluted 1:25,000 in 2% BSA/PBS. Plates were incubated with detection antibody or 1 h at 37°C before again washing three times with PBST. Reactions were developed with 100 μl/well TMB substrate (Supersensitive 1-component HRP substrate, BioFX, Owing Mills, MD, USA, Catalog # TMBS-0100-01) at room temperature for approximately 4 min. Reactions were stopped by addition of 100 μl/well 0.25 M sulfuric acid, and absorbance was measured at 450 nm using a Spectramax spectrophotometer (Molecular Devices, Sunnyvale, CA, USA). The standard curve was used to calculate serum concentrations of DX-2504.

### Experiment 2: IgG catabolism and pharmacokinetics of anti-FcRn antibody DX-2504 in cynomolgus monkeys

The study included 15 female cynomolgus monkeys from the New Iberia Research Center (NIRC) colony which were between 2.5 and 3.55 kg body weight. The study was conducted under an approved NIRC IACUC protocol at NIRC in New Iberia, LA, USA and performed in compliance with the protocol and UL Lafayette-NIRC Standard Operating Procedures. There were three monkeys per dose group, which included animals treated with IV vehicle (PBS, pH 7.2) or with DX-2504 (11.6 mg/ml) in PBS (pH 7.2) at 5 mg/kg (IV), 20 mg/kg (IV), 5 mg/kg (SC), or 20 mg/kg (SC). Each animal received in a volume of 1.72 ml/kg (in a peripheral vein IV and in the dorsal-scapular region SC) a dose on day 0 and on day 7 and blood samples were collected for serum approximately 30 min before each dose and then at 0.25, 2, 8, 12, 24, 48, 72, 96, 120, 144, and 168 h after each dose of DX-2504. IgG, IgA, IgM, and albumin concentrations in serum were measured using nephelometry and DX-2504 in serum was measured by ELISA.

#### Nephelometric measurement of immunoglobulin and albumin in serum from cynomolgus monkeys

Serum IgG, IgA, IgM, and albumin were measured in monkey serum using a BNII nephelometer (Siemens, Malvern, PA, USA). Serum samples were loaded into the instrument, which diluted each sample individually; IgG and albumin samples were diluted a minimum of 1:400 and the IgA and IgM a minimum of 1:20. For each test (IgG, IgA, IgM, or albumin), the diluted sample was mixed with an antiserum specific to the particular test and the amount of precipitation was measured by detecting the amount of light scattering of the particles. The results, expressed in milligram per milliliter, are calculated from standard curves using known calibrator reagents.

#### Measurement of anti-FcRn antibody DX-2504 in serum from cynomolgus monkeys

DX-2504 in serum was measured using a species-specific IgG ELISA. All unknown samples, quality controls (QCs), and standards were tested in duplicate wells. Briefly, 96 well plates (Costar, Catalog # 3369) were coated with 100 μl/well of goat anti-human IgG (H + L chain) monkey pre-absorbed (Bethyl Labs, Montgomery, TX, USA, Catalog # A80-319A) at a concentration of 1 μg/ml in 50 mM carbonate/bicarbonate pH 9.6 buffer solution (Sigma, St. Louis, MO, USA, Catalog # C3041-100CAP) and incubated at 37°C for 1 h (light plate shaking setting). After 1 h, plates were washed manually three times with 200 μl of 1×PBS supplemented with 0.5% Tween-20 (0.5% PBST) before they were blocked (200 μl/well) with 3% BSA (Jackson ImmunoResearch, West Grove, PA, USA, Catalog # 001-000-173) in PBS (Gibco, Grand Island, NY, USA, Catalog # 10010-023) and incubated for 1.5 h at 37°C (light plate shaking setting). Once plates were blocked, blocking solution was removed and the plate was blotted dry on paper towels. The standard curve was generated using DX-2504 (stock conc. 11.6 mg/ml) prepared in 100% pooled plasma from untreated monkeys, and then diluted serially twofold with pooled monkey plasma, starting from 1000 to 7.81 ng/ml. Each standard was then diluted 10-fold in 3% BSA/PBS to make a final standard curve with concentrations ranging from 100 to 0.78 ng/ml. QC samples were also included on the plates and prepared in the same manner as the standards. We included three QC samples corresponding to the expected ranges on the standard curve: high (80 ng/ml); middle (15 ng/ml), and low (4 ng/ml). Unknowns, QC, and standards (100 μl/well) were incubated at 37°C for 1 h (no plate shaking). Plates were then washed manually four times (200 μl/well) with 0.5% PBST before adding 100 μl/well of goat anti-human IgG (H + L chain)-HRP conjugated (Bethyl Labs, Montgomery, TX, USA, Catalog # A80-319P-8) diluted 1:25,000 in 3% BSA/PBS. Plates were incubated with detection antibody for 1 h at 37°C before washing four times manually with 0.5% PBST. Reactions were developed with 100 μl/well TMB substrate (Neogen Labs Enhanced K-blue, Catalog # 308177) at room temperature in the dark (no plate shaking) for approximately 10 min. Reactions were stopped by addition of 100 μl/well of 0.25 M sulfuric acid. Absorbance at 450 nm was monitored using a Spectramax spectrophotometer (Molecular Devices, Sunnyvale, CA, USA). The standard curve (four parameter logistical curve fit) was then used to calculate the serum concentration of DX-2504 vs. time.

### Experiment 3: Effect of anti-FcRn antibody DX-2504 on humoral immune response to model antigens keyhole limpet hemocyanin and tetanus toxoid in cynomolgus monkeys

The study included 18 naïve cynomolgus monkeys (9 males, 9 females) from the NIRC colony which were between 2.5 and 5.95 kg body weight. The study was conducted under an approved NIRC IACUC protocol at NIRC in New Iberia, LA, USA and performed in compliance with the protocol and UL Lafayette-NIRC standard operating procedures.

There were three study groups with three male and three female cynomolgus monkeys per group. Group 1 served as the control group (Vehicle, SC, dose volume of 1.75 ml/kg). On day 0, Group 1 animals were immunized with tetanus toxoid (TT) in the quadriceps muscle in 0.1 ml/kg. On day 28, Group 1 animals were dosed with Vehicle followed by keyhole limpet hemocyanin (KLH) in 0.5 ml (in the contralateral quadriceps muscle) and TT immunizations as above. Additionally, Group 1 was dosed with Vehicle on days 35 and 42. Group 2 received the same immunizations as Group 1, but was also administered DX-2504 (11.46 mg/ml) at 20 mg/kg SC on days 28 (prior to immunization with KLH and TT), 35, and 42. Group 3 received the same immunization treatment as Group 1, but DX-2504 was administered at 20 mg/kg SC on days 31, 38, and 45. DX-2504 was administered in the dorsal-scapular region in a volume of 1.75 ml/kg. A recovery period was included after the last dose of DX-2504 (25–28 days post-dose) to allow for blood sampling for serum in order to monitor the recovery of serum IgG levels. Blood was collected for serum (always prior to dosing) on days 0, 3, 7, 10, 14, 17, 21, 24, 28, 31, 35, 38, 42, 45, 49, 52, 59, and 70. Serum IgG, IgA, IgM, and albumin were measured in monkey serum using a BNII Nephelometer (Siemens, Malvern, PA, USA) as described. Animals received multiple vaccinations with TT prior to the start of the study and therefore use of this antigen was to model a secondary immune response. KLH was a novel antigen and used to model a primary immune response.

Keyhole limpet hemocyanin was (Imject^®^ mcKLH, Pierce, Rockford, IL, USA, Catalog # 77600) reconstituted with 1 ml of Sigma Water (Double Processed, sterile filtered, Catalog # W3500) to make a 20 mg/ml solution. TT vaccine (Super-Tet^®^ with Havlogen^®^; manufactured by Intervet/Schering-Plough Animal Health, supplied by Butler Animal Health) was stored and prepared for intramuscular injection as specified by the manufacturer.

#### Measurement of anti-KLH antibodies in serum from non-human primates

Antibodies against KLH were quantified using kits that measure either monkey anti-KLH IgM (Monkey Anti-KLH IgM ELISA Kit, Life Diagnostics, Catalog # 4000-4) or monkey anti-KLH IgG (Monkey Anti-KLH IgG ELISA Kit, Life Diagnostics, Catalog # 4010-4) as per the instructions of the manufacturer. For both assay kits, the levels of monkey anti-KLH antibodies are reported in nominal units derived from standard curves generated using calibrated standards.

#### Measurement of anti-TT antibodies in serum from non-human primates

Antibodies against TT were quantified using kits that measure either monkey anti-TT IgM (Monkey Anti-TT IgM ELISA Kit, Life Diagnostics, Catalog # 4300-4) or monkey anti-TT IgG (Monkey Anti-TT IgG ELISA Kit, Life Diagnostics, Catalog # 4310-4) as per the instructions of the manufacturer. For both assay kits, the levels of monkey anti-TT antibodies are reported in nominal units derived from standard curves generated using calibrated standards.

### Experiment 4: A pharmacologic comparability and toxicokinetic study of anti-FcRn antibodies DX-2504 and DX-2507 in cynomolgus monkeys

Six female cynomolgus monkeys weighing 3.2–3.4 kg originally sourced from Primate Products, Inc. (Miami, FL, USA) were transferred to the WIL Research Laboratories, LLC (Ashland, OH, USA) stock colony. Animals were maintained in accordance with the “Guide for the Cafe and Use of Laboratory Animals” (National Research Council, 1996). All monkeys were provided with an enriched environment in accordance with the WIL Research SOPs, and the Animal Welfare Act (9 CFR Part 3). The facilities at WIL Research Laboratories, LLC are fully accredited by the Association for Assessment and Accreditation of Laboratory Animal Care International (AAALAC International).

DX-2504 (18.2 mg/ml) or DX-2507 (34.6 mg/ml) in citrate–phosphate–trehalose buffer (100 mM citrate–phosphate buffer, 50 mM NaCl, 2% Trehalose, 0.01% Tween^®^ 80, pH 6.0) was administered via single SC injections on study days 0 and 7 to two groups (Groups 1 and 2) of female cynomolgus monkeys. Dose levels were 20 mg/kg DX-2504 and 20 mg/kg DX-2507. Each group consisted of three females.

The animals were observed twice daily for mortality and moribundity. Clinical examinations were performed daily and detailed physical examinations were performed approximately weekly. Individual body weights were recorded at least once during the pretest period, prior to dosing on study days 0 and 7, and approximately weekly during the recovery period. Blood samples for toxicokinetics and immunoglobulin assessments were collected from all animals prior to dosing and at approximately 2 and 12 h post-dosing on study days 0 and 7. On study days 1, 2, 3, 4, 5, and 6, blood samples for toxicokinetics and immunoglobulin assessments were collected from all animals based on the time of dose administration on study day 0 (±2 h). On study days 8, 9, 10, 11,12, 13, 14, 17, 21, 24, 28, 31, and 35, blood samples for toxicokinetics and immunoglobulin assessments were collected from all animals based on the time of dose administration on study day 7 (±2 h). Following the final blood collection for toxicokinetics and immunoglobulin assessments, all animals were returned to the colony (study day 35).

## Results

### Discovery of anti-FcRn antibodies by antibody phage display and affinity maturation

One-hundred sixty one unique phagemids that passed the secondary ELISA screening had distinct heavy chains. All 161 unique phagemids were subcloned and expressed as sFabs and screened in a FACS blocking assay of IgG-Fc to FcRn on 293C11 cells. This blocking assay yielded eleven sFabs with antagonistic anti-FcRn properties. The 11 sFabs were sequenced and reformatted to IgG1, AZ allotype, and further characterized for hFcRn binding by SPR analyses, which identified M90-F11 as the candidate with the highest affinity.

Parental clone M90-F11 was germlined and reformatted to F-allotype and designated DX-2500. Biacore SPR analysis of DX-2500 indicated unacceptable rapid *k*_off_ rates at both pH 6.0 and pH 7.4 [*k*_off_ (/s) = 1.77 × 10^−2^ and 7.52 × 10^−2^, respectively]. To improve the *k*_off_ rate, the germlined M90-F11 parental was affinity matured and a second selection campaign was conducted against hFcRn. Twenty-four sFab clones were sequenced, purified, shown to block IgG binding in FACS analysis, ranked in FcRn binding affinity by SPR analysis, and reformatted to IgG. Four IgG clones were selected for additional *in vitro* and *in vivo* testing. Based on *in vitro* and *in vivo* test results, plus expression and purification performance, clone M161-B04 was chosen as the lead antibody and was designated DX-2504 for further *in vivo* development.

DX-2504 has an unpaired cysteine residue in LC-CDR3 which was conserved during germlining. A free cysteine can result in disulfide bond shuffling, oxidation, and disulfide-linked aggregation impacting the antibody manufacturing process and product stability (54, 55). To mitigate this risk, the free cysteine in the LC-CDR3 in DX-2504 was mutated to an alanine. In addition, a C-terminal lysine residue was removed to eliminate charge variants derived from lysine cleavage to generate the variant of DX-2504 known as DX-2507.

Biacore analysis of hFcRn interaction with DX-2507 at pH 6.0 and pH 7.5 revealed that the kinetic constants for the interactions of the variants were essentially identical to those determined for DX-2504. The bioactivity of DX-2507 was further compared to DX-2504 in pharmacologic and toxicokinetic studies in cynomolgus monkeys. Table 1 provides the kinetic constants for the interaction of hFcRn with DX-2504 and DX-2507, and the resulting equilibrium dissociation constants (*K*_d_).

**Table 1:** **Kinetic constants for interaction of DX-2504 and DX-2507 with FcRn**.

	DX-2504			DX-2507		
pH	*k*_on_ (M^−1^s^−1^)	*k*_off_ (s^−1^)	*K*_d_ (nM)	*k*_on_ (M^−1^s^−1^)	*k*_off_ (s^−1^)	*K*_d_ (nM)
6.0	2.4 × 10^5^	3.5 × 10^−4^	1.5	1.3 × 10^5^	2.4 × 10^−4^	1.8
7.5	1.5 × 10^5^	2.8 × 10^−4^	1.9	1.0 × 10^5^	2.4 × 10^−4^	2.0

### IgG catabolism and pharmacokinetics of DX-2504 in TG32B mice (Experiment 1)

**Figure 1:**
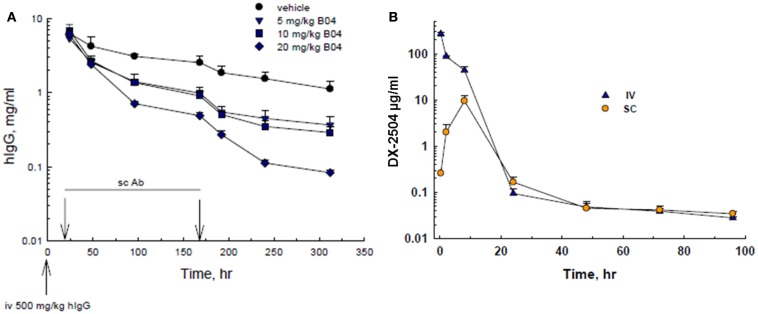
**IgG catabolism and pharmacokinetics of DX-2504 in TG32B mice**. **(A)** Dose response of IgG catabolism after 5, 10, or 20 mg/kg DX-2504 administered subcutaneously to TG32B mice at 24 and 168 h. **(B)** Pharmacokinetics of DX-2504 in TG32B mice after a single 10 mg/kg intravenous (IV) or subcutaneous (SC) dose. Error bars represent standard deviation.

Since the phage screening was directed toward human-FcRn, mouse experiments were conducted in human transgenic mice (TG32B). Furthermore, since endogenous mouse IgG does not recycle effectively through human-FcRn, human IgG was administered to the transgenic mice to serve as a model for IgG catabolism and FcRn antagonism. Therefore, transgenic mice were pre-treated with an injection of hIgG, followed by SC administration of 5, 10, or 20 mg/kg DX-2504. A rapid and dose-dependent acceleration of the catabolism of serum hIgG was observed as compared to the control animals (Figure 1A). A similar reduction in hIgG was observed following IV injection of DX-2504 (data not shown).

The pharmacokinetics of DX-2504 (10 mg/kg) following IV and SC administration was examined. As shown in Figure 1B, DX-2504 levels dropped rapidly, becoming almost undetectable after ~24 h following either SC or IV administration. In contrast, hIgG possesses a prolonged pharmacokinetic profile as shown in Figure 1A, with an approximate half-life of greater than 100 h.

### IgG catabolism and pharmacokinetics of DX-2504 in cynomolgus monkeys (Experiment 2)

Based upon results in the TG32B mouse model, an initial evaluation of pharmacology in non-human primates was performed to investigate the effect of DX-2504 on the catabolism of endogenous IgG.

**Figure 2:**
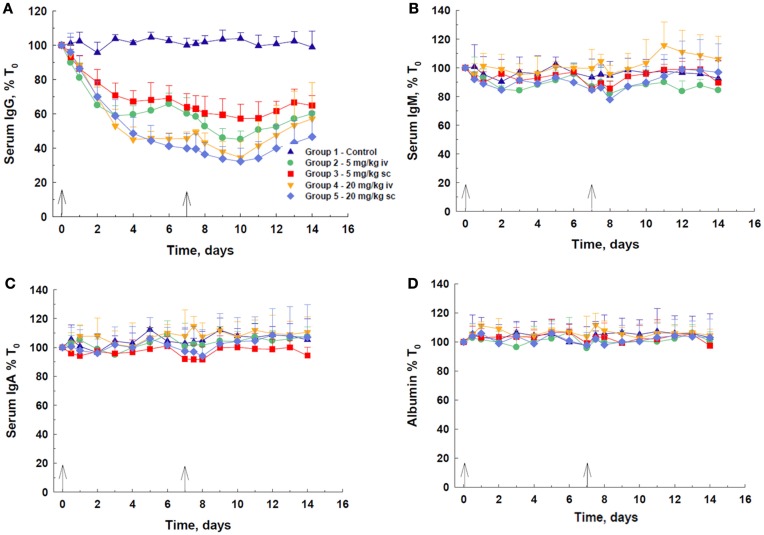
**IgG catabolism and pharmacokinetics of DX-2504 in cynomolgus monkeys**. Dose-dependent reductions in **(A)** total serum IgG but not **(B)** IgM, **(C)** IgA, or **(D)** albumin following DX-2504 in cynomolgus monkeys. DX-2504 was administered either intravenously (IV) or subcutaneously (SC) on days 0 and 7 (black arrows) at doses of 5 or 20 mg/kg. Data presented as percentage of baseline (day 0). Error bars represent standard deviation.

Following IV or SC administration of DX-2504, a decrease in total serum IgG concentration was observed at all doses (Figure 2A). A dose-dependent response was observed, with 20 mg/kg, via either route, being more effective than 5 mg/kg. At 20 mg/kg, there was not a discernible difference in the efficacy of DX-2504 given by either route of administration. The IV dose of 5 mg/kg DX-2504 appears more effective than the same SC dose. The maximum effect on IgG, i.e., ~60–68% depletion, was achieved by day 10, although most of the decrease was observed by day 7, after the first 20 mg/kg dose. Recovery of IgG levels began 3–4 days after the second dose. The serum concentrations of IgM, IgA, and albumin remained approximately the same throughout the 2 weeks of the experiment, although minor fluctuations were noted (Figures 2B–D).

**Figure 3:**
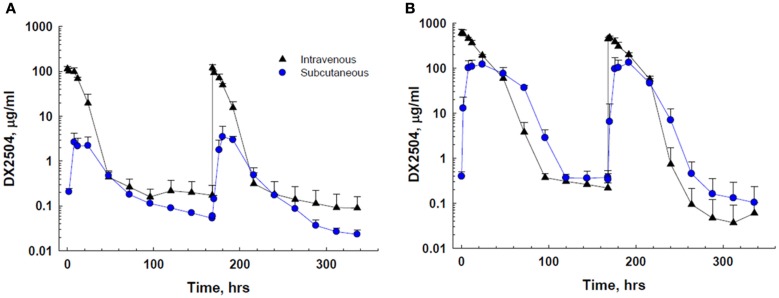
**Serum concentration of DX-2504 following intravenous or subcutaneous administration in cynomolgus monkeys**. **(A)** Serum concentration following 5 mg/kg DX-2504. **(B)** Serum concentration following 20 mg/kg DX-2504. DX-2504 was administered on days 0 and 7. Error bars represent standard deviation.

DX-2504 concentrations were measured in serum from all animals (Figure 3). Pharmacokinetic analysis was performed following each dose and results are presented in Table 2. Similar to the results from transgenic mice, DX-2504 rapidly disappeared from the circulation with a half-life less than 24 h following all doses and routes of administration.

**Table 2:** **DX-2504 pharmacokinetic parameters by dose following intravenous (IV) and subcutaneous (SC) administration to cynomolgus monkeys**.

Dose (mg/kg)	Route/day	*C*_max_ (μg/ml)	AUC (μg/ml × h)	*t*_1/2_ (h)
5	IV Day 0	114	1956	4.9
	IV Day 7	120	1556	4.8
5	SC Day 0	2.7	95	13.3
	SC Day 7	3.5	112	14.3
20	IV Day 0	596	13041	6.6
	IV Day 7	475	11613	5.2
20	SC Day 0	121	6372	7.2
	SC Day 7	132	5000	7.2

### Effect of DX-2504 on primary and secondary immune responses in cynomolgus monkeys (Experiment 3)

Two model antigens, KLH and TT, known to elicit significant IgG and IgM responses in cynomolgus monkeys were used to assess the primary and secondary immune response in the presence of DX-2504. The experiment was designed to examine the primary immune response to KLH and the secondary immune response to TT. Table 3 provides an overview of the study design. DX-2504 was administered SC either on the same day as the two antigens (second administration of TT and first administration of KLH) or 3 days following administration of the antigens.

**Table 3:** **Study design to evaluate primary and secondary immune response of cynomolgus monkeys following subcutaneous administrations of 20 mg/kg DX-2504**.

Group	Day 0	Day 28	Day 31	Day 35	Day 38	Day 42	Day 45
1	TT	Vehicle		Vehicle		Vehicle	
		TT	
		KLH	
2	TT	DX-2504		DX-2504		DX-2504	
		TT	
		KLH	
3	TT	TT	DX-2504		DX-2504		DX-2504
		KLH	

*TT, tetanus toxoid; KLH, keyhole limpet hemocyanin*.

**Figure 4:**
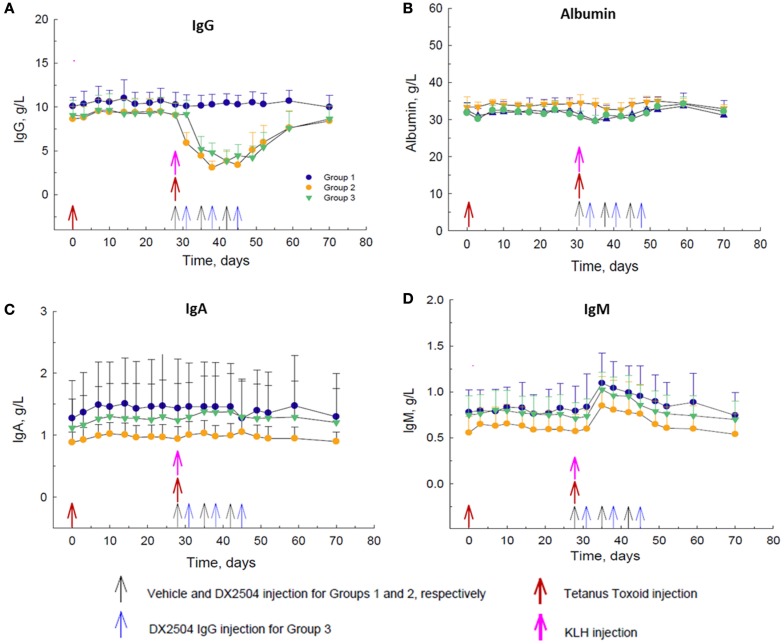
**Effect of DX-2504 in cynomolgus monkeys on primary immune response to keyhole limpet hemocyanin (KLH) and secondary immune response to tetanus toxoid (TT)**. Total serum **(A)** IgG, **(B)** albumin, **(C)** IgA, and **(D)** IgM. Groups 1–3 received TT on day 0 and 28 (red arrows) and KLH on day 28 (pink arrow). Group 1 received vehicle on days 28, 35, and 42 (black arrows). Group 2 received 20 mg/kg DX-2504 on days 28, 35, and 42 (black arrows). Group 3 received 20 mg/kg DX-2504 on days 31, 38, and 45 (blue arrows). Error bars represent standard deviation.

DX-2504, administered at 20 mg/kg SC on a once weekly schedule for 3 weeks (Groups 2 and 3), caused a decrease in total serum IgG concentrations of approximately 60–65% after the second dose (Figure 4A). DX-2504 had no effect on total serum concentrations of albumin (Figure 4B) or IgA (Figure 4C). There was a noticeable increase in total serum IgM concentration by day 35, 1 week after the administration of KLH consistent with an immune response to neo-antigens (Figure 4D). As expected, this increase in IgM was not affected by DX-2504 given on the same day as the antigens (Group 2, day 28) or 3 days after the antigens (Group 3, day 31).

**Figure 5:**
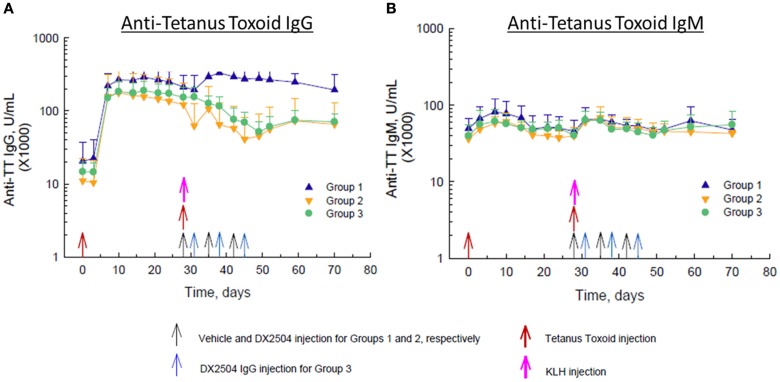
**Effect of DX-2504 on IgG and IgM immune response to tetanus toxoid (TT)**. **(A)** Antigen-specific IgG upon TT re-challenge and **(B)** IgM response. Groups 1–3 received TT on day 0 and 28 (red arrows) and keyhole limpet hemocyanin (KLH) on day 28 (pink arrow). Group 1 received vehicle on days 28, 35, and 42 (black arrows). Group 2 received 20 mg/kg DX-2504 on days 28, 35, and 42 (black arrows). Group 3 received 20 mg/kg DX-2504 on days 31, 38, and 45 (blue arrows). Error bars represent standard deviation.

Administration of TT on day 0 caused a large increase in anti-TT IgG antibodies in all animals by day 10 and a smaller increase in anti-TT IgG antibodies in control animals (Group 1) after the second injection of TT on day 28 (Figure 5A). Administration of DX-2504 on the same day as TT (day 28, Group 2) or 3 days after TT (day 31, Group 3) resulted in a reduction in anti-TT IgG antibodies of ~50–60% (Figure 5A). Both schedules for DX-2504 administration appeared to almost completely eliminate the anti-TT IgG responses. There was also an increase in anti-TT IgM antibodies after the first and second administrations of TT (Figure 5B). Administration of DX-2504 caused no change in the anti-TT IgM antibody responses, as expected (Figure 5B).

**Figure 6:**
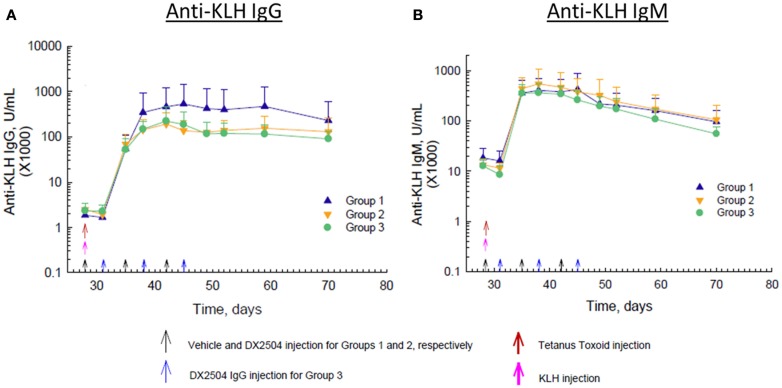
**Effect of DX-2504 on IgG and IgM immune response to keyhole limpet hemocyanin (KLH)**. **(A)** IgG and **(B)** IgM to novel antigen KLH. Groups 1–3 received tetanus toxoid on day 0 and 28 (red arrows) and KLH on day 28 (pink arrow). Group 1 received vehicle on days 28, 35, and 42 (black arrows). Group 2 received 20 mg/kg DX-2504 on days 28, 35, and 42 (black arrows). Group 3 received 20 mg/kg DX-2504 on days 31, 38, and 45 (blue arrows). Error bars represent standard deviation.

Immunization with KLH on day 28 caused a marked rise in anti-KLH IgG antibodies within about 7 days and peak levels of antibodies occurred by day 14 after immunization (Figure 6A). Administration of DX-2504 inhibited the rise in anti-KLH IgG antibodies, resulting in markedly lower antibody levels in Groups 2 and 3 compared to Group 1 (Figure 6A). Anti-KLH IgG antibody levels were the same on day 35, but by day 38, a difference in the levels was observed. Anti-KLH IgM antibody levels also rose sharply after immunization, but administration of DX-2504 had no effect on these levels (Figure 6B).

### Pharmacologic comparability and toxicokinetics of DX-2504 and DX-2507 in cynomolgus monkeys (Experiment 4)

*In vitro* and *in vivo* studies revealed DX-2504 to be a potent and specific antagonist of human-FcRn. It effectively increased the catabolism of exogenously administered IgG in mice and reduced IgG levels in non-human primates with no apparent effects on IgM, IgA, or albumin in non-human primates. However, as noted, there was concern that an unpaired cysteine residue in DX-2504 could impact antibody manufacturing and product stability, thereby disrupting future clinical development. To mitigate this risk, DX-2507 was developed and the pharmacokinetics and pharmacodynamics of the molecule compared to that of DX-2504.

**Figure 7:**
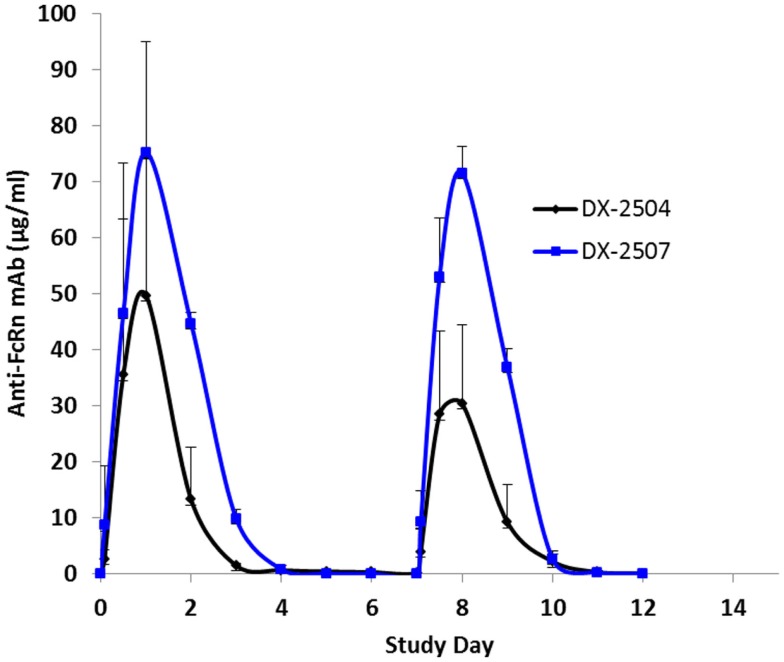
**Serum concentrations following subcutaneous (SC) administration of DX-2504 and DX-2507 to cynomolgus monkeys**. Animals received 20 mg/kg DX-2504 or DX-2507 on days 0 and 7. Error bars represent standard deviation.

Cynomolgus monkeys received two doses (once weekly, 20 mg/kg subcutaneous) of either DX-2504 or DX-2507. All animals survived to study termination. There were no clinical observations or effects on body weights noted for either treatment group (data not shown). DX-2504 serum concentrations were detected from 2 h post-dosing on study day 0 through study day 11 in two animals and on study day 13 in a single animal (Figure 7). DX-2507 serum concentrations were detected from 2 h post-dosing on study day 0 through study days 11, 12, and 17 in individual animals (Figure 7).

In general, the toxicokinetic parameters for each antibody were consistent on study days 0 and 7; the exception being the greater mean elimination *t*_1/2_ value for DX-2504 on study day 0 (Table 4). This is thought to be due to sustained serum concentration values ranging from 0.25 to 0.46 μg/ml (observed on study days 5 through 7). The overall exposure of DX-2507 was greater than that observed for DX-2504. The mean *C*max and AUClast values for DX-2507 on either study day 0 or 7 were between two to threefold greater than the values calculated for DX-2504. The difference in exposure is consistent with the greater reduction in IgG levels in animals that were administered DX-2507.

**Table 4:** **Mean (SD) toxicokinetic parameters following SC administration to cynomolgus monkeys**.

Test article	Study day	*C*_max_ (μg/ml)	*T*_max_ (day)	AUC (μg/ml × day)	CL/F (ml/day/kg)	Vz/F (ml/kg)	*t*_1/2_ (day)
DX-2504	Day 0	51.9 (25.8)	0.8	70.8 (32.2)	341.0 (204.5)	879.1 (407.0)	1.9 (0.2)
	Day 7	32.0 (15.7)	7.8	47.5 (20.0)	492.3 (264.0)	312.4 (252.0)	0.4 (0.1)
DX-2507	Day 0	75.3 (19.7)	1.0	135.6 (29.4)	152.0 (31.8)	74.1 (35.0)	0.3 (0.1)
	Day 7	71.6 (4.7)	8.0	120.3 (3.2)	166.3 (4.3)	73.6 (24.8)	0.3 (0.1)

**Figure 8:**
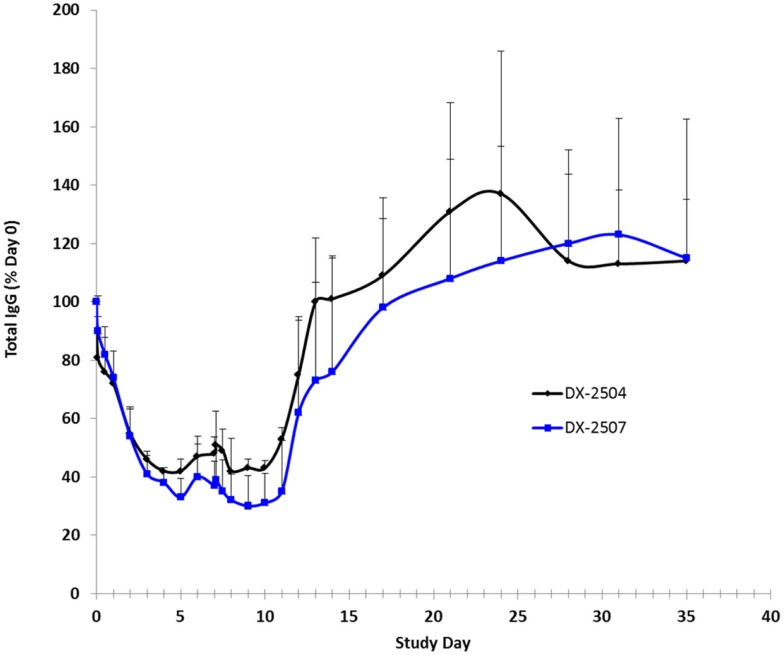
**Total IgG as a percentage of baseline (day 0) in cynomolgus monkeys following subcutaneous (SC) administration of DX-2504 or DX-2507**. Animals received 20 mg/kg DX-2504 or DX-2507 on days 0 and 7. Error bars represent standard deviation.

Cynomolgus monkey IgG levels were reduced following the administration of both DX-2504 and DX-2507 (Figure 8). Following administration on study day 0, mean total IgG levels were reduced to 42 and 33% of pre-dosing baseline levels in the DX-2504 and DX-2507 groups, respectively. Prior to administration on study day 7, mean total IgG levels increased to 48 and 37% of pre-dosing baseline levels in the same treatment groups. Following dose administration on study day 7, mean total IgG levels were reduced to 42% of pre-dosing baseline values in the DX-2504 group and to 30% of pre-dosing baseline values in the DX-2507 group. Total IgG levels returned to pre-dosing baseline values on study day 13 in the DX-2504-treated animals and on study day 21 in the DX-2507-treated animals.

## Discussion

Human IgG molecules are non-specifically taken up into the endosome where they bind to FcRn under acidic conditions and as such are effectively rescued from the endosomal degradation pathway. FcRn then recycles back to the cell surface where the antibodies are released in the neutral pH conditions. We therefore reasoned that an effective FcRn receptor antagonist would be one that not only competed for IgG binding but would remain bound throughout the cycle – both in the endosome at acidic pH and at the cell surface at neutral pH. Since FcRn is also responsible for the extended half-life of serum albumin, we also wanted an antibody that would effectively block IgG binding without disturbing albumin binding. Such a specific antibody is feasible since it has been demonstrated that IgG and albumin bind opposite faces of the FcRn molecule (32, 34, 56).

The context of FcRn as a membrane protein was an important consideration in the design of our antibody selection strategy. Since soluble domains, as compared to membrane bound forms, may impact the function of antibodies that result from display library selections, we opted to incorporate both the soluble form of the FcRn/β2M heterodimer and the membrane-bound form into the library selection strategy. ELISA-based screening approaches were then used to identify selectants that met the above criteria to move forward with additional characterization. Results of *in vivo* testing in animal models and manufacturing considerations then informed further optimization of candidates, leading to the development of DX-2504 and subsequently DX-2507.

Studies carried out in transgenic mice expressing hFcRn demonstrated that administration of DX-2504 led to increased hIgG catabolism. However, studies of hFcRn antagonism in rodents are of somewhat limited predictive value because of the lack of human IgG synthesis to counterbalance IgG degradation, as well as cross-species differences in FcRn reactivity (57). For this reason, we conducted additional studies in non-human primates. In agreement with findings from the transgenic mouse studies, IV or SC administration of DX-2504 to non-human primates was effective at lowering serum concentrations of IgG by approximately 60%. DX-2504 had no effect on levels of total IgM, IgA, or albumin. IgM and IgA do not bind FcRn and therefore one would not expect an effect on levels of these two immunoglobulins. However, albumin does bind to FcRn and the lack of an effect on levels of albumin is a testament to the specificity of DX-2504 and provides insight into the location of the antibody epitope.

There was a clear dose dependency to the depletion of IgG in monkeys, with 20 mg/kg, either IV or SC, being more effective than 5 mg/kg by either route. At 20 mg/kg, there was no discernable difference between IV and SC dosing. This is important because it could allow for SC administration in the clinical setting. Similar to the short half-life of DX-2504 observed in transgenic mice, DX-2504 rapidly disappeared from the circulation of non-human primates. It is conceivable that the FcRn receptor occupancy of DX-2504 prevents accurate clearance estimates from serum drug levels.

Results from the examination of DX-2504 on the primary and secondary immune response in non-human primates were in line with expectations for treatment with an anti-FcRn antibody. Total IgG levels decreased rapidly after administration, while concentrations of albumin, IgM, and IgA were unchanged. Anti-KLH and anti-TT IgG antibodies were lowered to approximately the same level as total IgG levels (50–70% reduction) when compared to control animals.

The immune response to KLH was robust, as expected, and only the anti-KLH IgG antibodies were affected by treatment with DX-2504; anti-KLH IgM antibodies were unchanged. It is also of note that the anti-KLH IgG response was initially robust, but eventually markedly reduced by DX-2504 treatment. Taken together, the results suggest that DX-2504 has a specific effect on IgG type immunoglobulins but does not interfere with other classes of immunoglobulins and the humoral antibody response to novel antigens. Thus, FcRn antagonism represents a promising treatment strategy for autoantibody-mediated autoimmune diseases by targeting the IgG autoantibody, the end point of the pathogenic humoral immune reaction without significantly suppressing normal immune function.

While DX-2504 possessed several of the characteristics important to the development of a therapeutic antibody targeting FcRn, there was concern over its manufacturability due to an unpaired cysteine in LC-CDR3. The ability to produce and maintain the tertiary and quaternary structure of a human therapeutic antibody through manufacturing to storage is a key consideration in translating novel discoveries into therapeutic strategies in clinic. To mitigate the risk of the unpaired cysteine disrupting the advancement of the molecule in clinical development, variants of DX-2504 were created. DX-2507, a variant in which the free cysteine was changed to an alanine, bound to FcRn with similar affinity to that of DX-2504. It was well-tolerated and possessed a slightly improved pharmacokinetic profile with overall exposure two to threefold greater than that observed for DX-2504 in non-human primates. DX-2507 reduced total IgG levels by >60% following two subcutaneous injections and levels returned to pre-dose baseline values approximately a week following the second injection.

Interestingly, the terminal half-life of both DX-2504 and DX-2507 following either IV or SC administration to non-human primates was on the order of hours but resulted in prolonged reductions in serum IgG (~1 week). The high affinity of the antibodies for FcRn at neutral pH may provide a possible explanation for this apparent lack of a correlation between pharmacokinetic and pharmacodynamic activities. Endogenous IgG has limited affinity for FcRn at physiological pH and therefore dissociates when recycled back to the cell surface following binding to FcRn in the endosome. Estimates for the binding of endogenous IgG via Fc domains range from low nanomoles at pH 6 and negligible to >1 μM at pH 7.4 (58, 59). In contrast, the high affinity of the engineered antibodies supports the concept that they remain largely bound to the receptor upon exocytosis to the cell surface. Indeed, Biacore studies demonstrated that DX-2504 and DX-2507 bind to hFcRn with high affinity at both pH 6.0 (*K*_d_ = 1.5 and 1.8 nM, respectively) and pH 7.4 (*K*_d_ = 1.9 and 2.0 nM, respectively). Thus, the high affinity at pH 7.4 may prevent it from following the natural recycling pathway which typically provides IgG their long serum half-lives *in vivo*. Alternatively, we cannot rule out the possibility of irreversible binding of the FcRn-inhibitors to FcRn *in vivo*. To date, we have not generated experimental data in support of either possibility.

Recognition of the importance of FcRn in mediating IgG homeostasis represents a promising therapeutic strategy for the treatment of antibody-mediated autoimmune diseases. FcRn antagonism may also be a compelling strategy for the management of neutralizing anti-drug antibodies associated with biologic replacement therapies, such as those observed with replacement proteins for hemophilia and lysosomal storage diseases. The antibodies described are capable of reducing total and antigen-specific IgG >60% *in vivo*, which is in line with data suggesting that clinical improvements can be achieved in several autoimmune diseases when autoantibody levels are reduced >50% (60–62). Importantly, FcRn antagonism does not totally abolish serum IgG which reduces the risk of hypogammaglobulinemia. The lack of an effect on other classes of antibodies, as well as the primary response to novel antigen, also suggests FcRn antagonism may result in less immune system disruption and less infection risk. Ultimately, the safety of an anti-FcRn therapeutic approach can only be born out in human clinical trials but these data provide important proof of concept and support the viability of the approach.

## Conflict of Interest Statement

Andrew E. Nixon, Jie Chen, Chris TenHoor, Malini Viswanathan, Daniel J. Sexton, and Joseph Biedenkapp are currently full-time employees of Dyax Corp. At the time the research was conducted, Bob Ladner, Arumugam Muruganandam, Diana Martik, Dina Wassaf, and Clive Wood were full-time employees of Dyax Corp.; Jennifer Dumont, Liming Liu, Adam Mezo, and Alan J. Bitonti were full-time employees of Syntonix Pharmaceuticals, a wholly-owned subsidiary of Biogen Idec.
